# City of Detroit, Hamtramck, Highland Park, and School of Choice: School Aged Environmental Risk Exposures

**DOI:** 10.1007/s11524-025-01035-1

**Published:** 2025-12-06

**Authors:** Heather Moody, Sue C. Grady, Lelia Sigmon

**Affiliations:** 1 Department of Geography and Sustainable Planning, Grand Valley State University, 1 Campus Drive, Allendale, MI 49401 USA; 2 Department of Geography, Michigan State University, 673 Auditorium Rd., Rm 207, East Lansing, MI 48933 USA

**Keywords:** School of choice, Children’s health, Environmental hazards, Segregation, Site selection, Detroit, Michigan

## Abstract

This research studies the levels of environmental pollutants related to four different school of choice types in the cities of Detroit, Hamtramck, and Highland Park in 2021. It is hypothesized that public and for-profit charter schools will be located in older buildings and in areas of greater pollution. Indicators of pollution exposure included Superfund and Brownfield sites, location and pounds of Toxic Release Inventory, and mean blood lead levels of the children, which were geospatially joined to a 1-mile buffer surrounding the four school of choice types within the study area. The age of the school buildings was examined to estimate the potential for internal toxic exposures. The spatial relationship between neighborhood racial segregation, poverty, and schools of choice types was also assessed. Within the study area, public and for-profit charter schools and their surroundings exert the potential for greater internal and external pollution exposure to children. For-profit charter schools were significantly the oldest, potentially exposing students to a variety of toxicants. All schools were located in racially segregated and relatively high-poverty neighborhoods, with public and for-profit schools exhibiting the highest poverty percentages. Public policy, such as siting guidelines and school building maintenance, should be used to decrease these potential exposures to hazards and avoid worsening existing inequalities. Addressing them is important to prevent further disadvantage to already vulnerable children.

## Introduction

Schools can be a significant factor in childhood exposure to both indoor and outdoor toxicants, especially given the hours students spend in their respective places. Urban settings experiencing disinvestment may contribute more interior and exterior pollutant sources due to older building structures and location next to industrial emissions and industrial waste sites [1].

In the United States, older school buildings may expose children to an increased risk of contact with a variety of pollutants. Exposure to lead-based paint dust and pipe water is most commonly associated with lower IQ scores, headaches, slowed growth, hearing problems, brain damage, nervous system disorders and behavior and attention problems. Mold from leaking, damp, or remnant sources has the potential to cause irritation of the eyes, skin, nose, throat, and lungs regardless of allergies and can provoke asthma attacks in children allergic to mold. Breathing asbestos-based insulation dust is commonly associated with asbestosis, lung cancer and mesothelioma. Exposure to polychlorinated bisphenols within older lighting ballasts/caulk is linked to cancer, reproductive effects and neurological effects to name a few. Carbon monoxide exposure results from improper ventilation of dated furnaces causing dizziness, fatigue, headaches, disorientation, nausea and at high levels of exposure, loss of consciousness and death [2].

Analyzing Michigan public schools, Kweon et al. [3] measured the distances from 3660 Michigan public schools to highways and industrial facilities. Schools located closer to pollution emissions had a higher percentage of Black, Hispanic, or economically disadvantaged children than White students.

The school of choice type (public, private, charter, for-profit charter) children attend may also be an indication of toxic exterior exposure. Using a Gaussian Process model for spatiotemporal regression of particulate matter (PM), Mullen et al. [4] found that charter schools exposed students to PM_2.5_ concentrations 20% higher than non-charter schools in Salt Lake County, Utah. Gulosino and Lubienski [5] found that in Detroit, charter schools tend to be located in vacant commercial/industrialized buildings. Further, Michigan conference proceedings [6] aimed to discover the relationship between polluted school environments and the cognitive development/functioning of school-aged children, especially for those in Detroit. There is a void in siting guidelines for new and existing schools and the greatest numbers of schools in Michigan are located in the more polluted parts of their cities; these are located in predominantly low-income/minority neighborhoods, especially in Detroit. It was reported that charter school sponsors might, as was the case with other case studies, choose to locate schools on/in less expensive sites of abandoned industrial and commercial facilities instead of reusing the many closed public-school buildings in Detroit. The conference proceeding research concluded that air pollution around all school types is linked to poorer student health and academic performance. Assessing internal and external air pollution sources or assessing soil contamination as in the Beard Elementary School built on a hazardous waste site, is needed [6].

In rust belt cities of the United States, an established relationship exists between the concentration and duration of the Black population and capital divestment and urban population decline [7]. These cities, especially those with supermajorities of Black populations such as Detroit, Cleveland, and Pittsburgh, have experienced policies of racial prejudice accompanied by a lack of housing supply [7], access to clean water [8], increased pollution [9], etc., all social determinants of health and wealth. Detroit lost over 1 million people from 1960 to 2020 [10] due to extreme economic decline and the movement of the auto and manufacturing industries to other states/countries as typical of other rust belt cities [7].

Another set of research questions addresses socioeconomic and race/ethnicity segregation in schools of choice. Nationally, Grineski and Collins [11] found that the proportion of Black students was the most significant predictor in enrollment to public urban schools with the highest neurotoxicant risk, controlling for socioeconomic status (reduced/free lunch). Nationally, charter school openings have been shown to locate in low-income areas of segregated minority communities [12–14]. More recently, Farmer et al. [15] spatially associated Chicago’s new charter schools with Black neighborhoods. Regarding Detroit, Green, Sanchez, and Castro [16] used hot-spot analysis to determine that charter schools opened in ZIP codes dominated by Black and Latino neighborhoods, especially for-profit charter schools. Here, public schools have closed at the expense of charters due most recently to ‘racist neoliberal policies’ such as Prop A, emergency management installation, and charter school policy promotion leading to the marketization of Detroit’s public school educational system [16–18]. As a policy tool to this marketization, Proposition A is a state law passed in 1994 that repealed property taxes as the source of public-school funding. It instead allocated funding to districts based on a tax-dependent, per-student basis, thus leaving poor districts less able to support their schools through increased taxes [16]. Another policy tool, the installation of emergency management (as opposed to democratically elected individuals) in 1999 and 2014 resulted in tremendous defunding of the public school system, allowing the market for charter schools to increase [16]. Compared to public schools, Gulosino and d’Entremont [5] found that in the State of New Jersey, racial segregation (%White versus %minority) is most severe surrounding charter schools’ neighborhoods (block groups). Lee [19] found that in Michigan, for-profit charter schools select sites as part of a critical factor in making a profit. Using logistic regression at the block-group level of analysis in the Detroit metropolitan area, Lee shows for-profit site selection preference is in school districts with greater proportions of African American and Hispanic populations [19].

This study seeks to spatially relate the four school of choice types to a variety of environmental indicators of pollution, segregation, and poverty in the cities of Detroit, Hamtramck, and Highland Park. Over the last decade, public education and its buildings have been divested or closed with the opening of for-profit charter schools [16–18]. From 1999 to 2016, 195 public schools closed and 116 charter schools opened in Detroit [16] and by 2017, 80% of charter schools in Michigan were operated by for-profit centers. This figure is only 16% nationwide [20].

This study includes the number and/or concentration of external pollutant sources/indicators using a 1609-m (1 mile) buffer surrounding each school of choice type. External indicators include Toxic Release Inventories (TRI), the number of Brownfield/Superfund sites, the mean of childhood blood lead levels (BLLs), and the age of the school building serving as a proxy for a myriad of potential internal exposures. Neighborhood segregation and poverty levels are also spatially related to school of choice type. Given the literature review, it is hypothesized that within the cities of Detroit, Hamtramck, and Highland Park, public schools and for-profit charters, will have older school buildings, be located next to industrial emissions, Superfund/Brownfield sites, higher mean childhood BLLs, and fall within impoverished Black–White segregated neighborhoods.

## Materials and Methods

The following defines the variables analyzed and methods/sources used to obtain them. The end of each section will include geospatial analysis, mapping, and statistical methods. All analyses were performed using SPSS Statistical Software 29.0.1.0. and mapping using ArcGIS Pro 3.1.

### Study Area and Population Vulnerability

This study was conducted in the cities of Detroit, Hamtramck, and Highland Park in southeast Michigan, USA. The city of Detroit’s boundary encompasses both Hamtramck and Highland Park, all situated within Wayne County. Detroit has a population of 620,376 and an area of 1337 square miles including Hamtramck and Highland Park. Hamtramck’s population is 27,834, and Highland Park’s is 8977 [21].

### School of Choice Type, Enrollment, and Buffer

All Detroit, Hamtramck, and Highland Park Public Community Schools including early childhood, elementary, middle school, high school, and enrollments were compiled from Michigan Department of Education’s District Homepages for 2021 [22–24]. Local Education Agency (LEA) Districts house the public school district but exclude powers over charter schools as defined in MCL 380.6 and 380.11a. Part 6 details the districts. Charter schools are public school academies operated by a local board or management company (Educational Management Organization) approved by a chartering agency under the provisions of the Michigan Revised School Code, Sect. 501–507 [25]. Charter schools are considered public schools and receive per-pupil funding but operate outside of the normal regulatory and accountability structures that govern the operation of traditional public schools. A charter school in Michigan is “a state-supported public school under the state constitution, operating under a charter contract issued by a public authorizing body” [26]. All Detroit, Hamtramck, and Highland Park Charter Schools including early childhood, elementary, middle school, high school, and enrollments were compiled from Michigan’s Charter School Association for 2021 [27]. From here, profit-driven charters versus not-for-profit charters were distinguished. For-profit charter schools are organizations that contract out some or all of their operations or services to a for-profit organization; however, for tax purposes, they are considered not-for-profit and the methodology for discerning between the two was adopted from the National Education Policy Center [28] and/or verified with the State of Michigan’s Department of Education “Public School Academy Districts with Education Service Providers and Chartering Agencies” list [29]. From 2018 to 2019, Michigan remained the state operating with the most for-profit charters with over 85% of its charter schools privately operated [29]. Private schools are not administered by a public (or LEA) school district, but may receive public school services as a private, denominational, or parochial school based upon Michigan Compiled Laws. MCL 380.5 (4). All Detroit, Hamtramck, and Highland Park private schools including early childhood, elementary, middle school, high school, and enrollments were compiled from The Michigan Department of Education’s Nonpublic and Home Schools website for 2021 [30]. Public schools were denoted as 1, private as 2, for-profit charters as 3, and not-for-profit charters as 4.

A buffer of 1609 m (1-mile) was established around each school location to capture the environmental variables spatially associated with and described below. The 1-mile distance was informed by Meng [31] and the hazards associated with proximity to TRI sites. The schools and buffers were geospatially joined with the sites, pounds of TRI, and average BLLs.

### Age of School Building as Proxy for Internal Exposures

A “year-built” variable was derived from the Michigan Department of Education for all public schools and some private schools. A Freedom of Information Act request was submitted to the Detroit Assessors’ office for property records for the remaining tax-exempt (charter and private) schools of choice. Some of these records were not made available. In these remaining cases, year-built was obtained from assessor property records by personal visits to the schools, subscribing to real estate associations, archival Detroit library departments, the Lansing Michigan Library, the Detroit assessor’s office, Public Works office—building department, Maps and Records Department, Land Use Zoning Department, and the Clerk’s office.

The Detroit Public Schools had their buildings assessed for safety, space, and capacity in 2018 as summarized in an Executive Summary Facilities Assessment and School Facility Planning, document [32]. They found that many buildings were recommended to conduct water quality tests on a regular basis as buildings constructed prior to 1985 may contain lead-based solder used for pipe joint union. Older buildings are also more likely to contain asbestos, molds, etc. as described in the document.

The year-built value was dichotomized by assigning a 1 to those constructed before 1985 and 0 if constructed on or after 1985.

### Toxic Release Inventory (TRI), Brownfield, and Superfund Sites

All three variables quantify and locate either existing pollutant releases to air, land and water, or are areas deemed to contain hazardous pollutants as defined and obtained from the US EPA or Michigan’s Environment, Great Lakes, and Energy (EGLE) websites for 2021. These locations were geocoded to the city of Detroit, Hamtramck, and Highland Park’s boundaries/addresses. The TRI data and locations were obtained from the EPA’s total release inventory [33] by state and included total pounds to air, land, and water. The number of sites within the 1-mile buffer was geospatially counted and then the average pounds of all three media were calculated. The Brownfield locations were geospatially counted in each buffer zone and obtained from Michigan’s State EPA, EGLE [34]. There was one Superfund site left in the study area (most became Brownfield sites) and its address was derived from the U.S. EPA’s National Priorities List and Superfund Sites website [35]. This one site was geospatially counted in relation to the buffer zones surrounding the schools.

### Blood Lead Levels (BLLs)

Childhood BLLs, especially higher values, indicate that the children are exposed to lead either in the home, school, or other locations where it is likely that lead pipes, paint, and external emissions exist. These levels were obtained from the Michigan Department of Health and Human Services (MDHHS) for the Detroit area for the years 2010–2020. Although all other variables used 2021 data, this 10-year span captures more data as not every child is blood lead tested every year. All duplicate test results of the same child over this period were removed, keeping only the highest BLL value. The children’s test results were de-identified integers in micrograms per deciliter (ug/dL) of blood and were accompanied by addresses geocoded to the cities of Detroit, Hamtramck, and Highland Park with a 99.58% match rate and a 0.004% tied rate resulting in a total of 156,346 single test results for the three cities. No records were unmatched.

Four separate attribute queries were made for each school of choice type, selecting mean BLL results in the 1-mile buffer around public, private, for-profit charter, and not-for-profit charter schools. This provided the total number of children living within 1-mile buffers around the four school of choice types in addition to the BLL ranges and averages of individual children.

### Neighborhood Segregation Indices

Point estimates from the Decennial Census 2020 [36] were used as input values to calculate the isolation index I at the census tract level as follows:

$$I= \sum_{j}\left[\left(\frac{{n}_{j}^{B}}{{N}^{B}}\right)-\left(\frac{{n}_{j}^{B}}{{n}_{j}}\right)\right]$$

where ${n}_{j}^{B}$ is the number of non-Hispanic Black residents in tract $j$, ${n}_{j}$ is the population size of tract $j$, and ${N}^{B}$ is the total number of Black residents across all census tracts. The value of *I* reflects the mean exposure of non-Hispanic Black residents to other non-Hispanic Black residents with the index, a probability, bounded by 0 and 1 (complete isolation of the group with its own members).

These variables were studied to discover the confounding and controlling nature of poverty, segregation, and the intersection of both related to potential pollutant exposure and school of choice type.

Percent poverty for Detroit’s census tracts was calculated using the ratio of family income to poverty level from the American Community Survey 2021 5-year estimate [33]. Poverty was defined as a less than 1.0 threshold divided by the total population of the census tract.

### Statistical Methods

A one-way ANCOVA, controlling for enrollment, was conducted using the dichotomous value of year-built (1, 0) compared to the four different school types. Another one-way ANCOVA, controlling for enrollment, was conducted between the four different school types and the mean BLLs within the buffer. This study is controlling for enrollment as a leveling tool for potential exposure risk given that some schools had very small numbers of children and others had much larger populations.

## Results

### Study Area and Population Vulnerability

When racial residential segregation was measured across the Detroit Metropolitan Area the cities of Detroit, Hamtramck, and Highland Park neighborhoods were highly segregated as defined by the isolation index—i.e., the likelihood that a Black person would only come into contact with another Black person (mean = 0.86, range 0.15–0.94). Across Detroit, Hamtramck and Highland Park neighborhoods, the mean poverty level was 34% (range 4.2 to 71.0%).

### School of Choice Type, Enrollment, and Buffer

There were 240 schools open and identified in 2021. Detroit had 106 public schools (46,054 enrolled), 27 private schools (3548 enrolled), 64 for-profit charter schools (24,261 enrolled), and 26 not-for-profit charter schools (10,467 enrolled). Hamtramck had eight public schools (3744 enrolled) and five for-profit charter schools (2553 enrolled) with zero private or not-for-profit charters. Notably, all of Highland Park’s public schools have transitioned into four for-profit charter schools (873 enrolled).

### Age of School Building as Proxy for Internal Exposures

In 2021, public schools had a year-built range of 1896–2012 and a mean of 1951; private schools a range of 1836–2001 and a mean of 1945; for-profit charters a range of 1886–2010 and a mean of 1941; not-for-profit charters a range of 2016–2000 and a mean of 1947. For-profit charter schools were significantly older than all other school of choice types followed by public schools then private.

The one-way ANCOVA met normality check assumptions. There was a significant difference in year-built between school of choice types [*F*(1, 237) = 3.986, *p* = 0.047] while controlling for enrollment. Post hoc tests showed that for-profit charters were built in significantly different years compared to public (*p* = 0.023) and private (*p* = 0.020) schools controlling for enrollment. From the adjusted means, for-profit charter schools were most related to year-built (*M* = 0.945) followed by not-for-profit charters (*M* = 0.923), private (*M* = 0.852), and then public schools (*M* = 0.816). The partial Eta squared of 1.7% indicates the variance in school of choice type is explained by the year-built (small effect) controlling for enrollment. These findings are not surprising given that in general, school buildings in Detroit are relatively dated.

### Toxic Release Inventory, Brownfield, and Superfund Sites

**Fig. 1:**
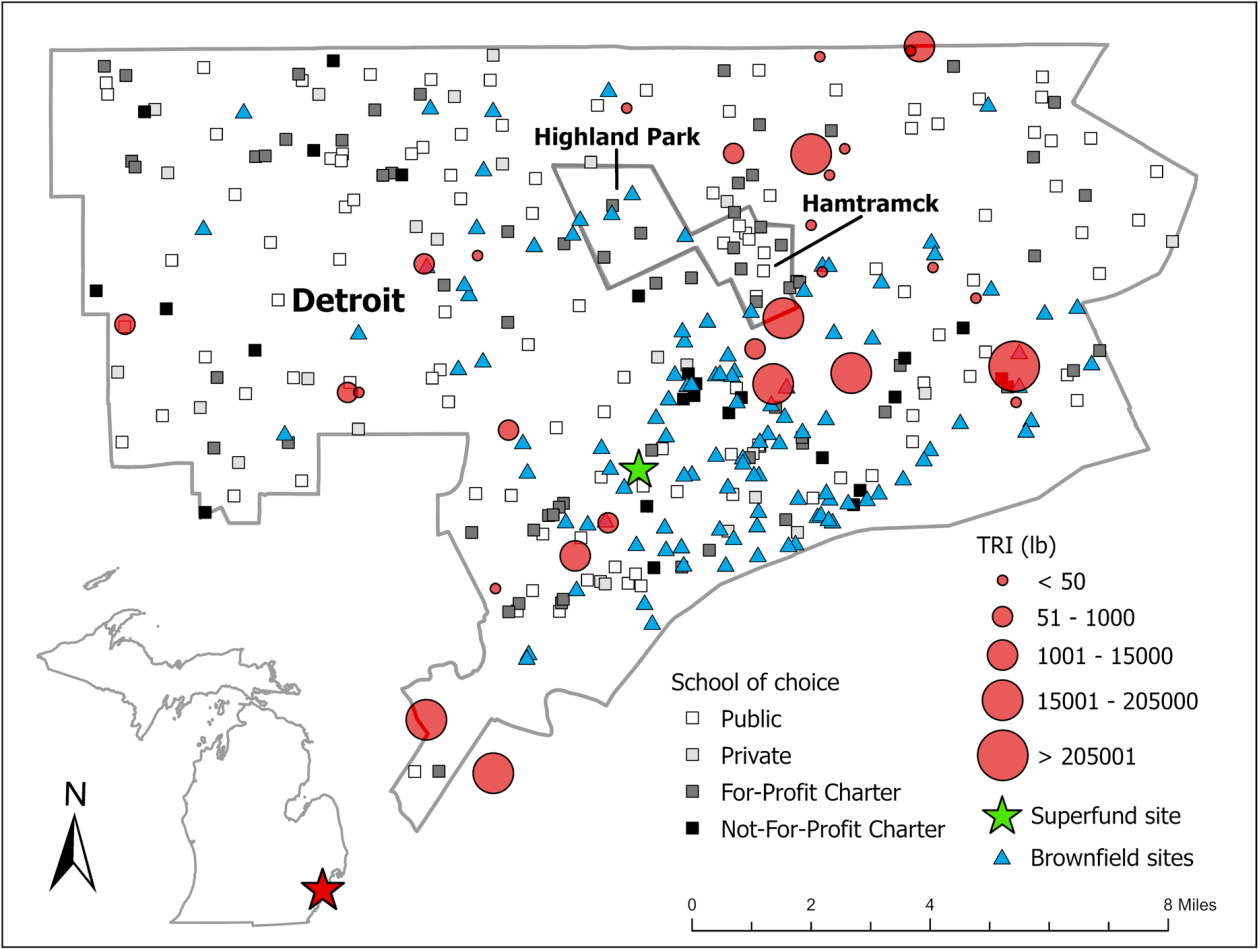
Toxic release inventory (TRI) sites and pounds, Superfund sites, and Brownfield sites with school of choice type (public, private, for-profit charter, and not-for-profit charter). The cities of Detroit, Hamtramck, and Highland Park

Figure 1 depicts each school of choice type with the locations of the TRI, Brownfield, and Superfund sites. Only one symbol each was used for multiple sites falling within each school buffer as to reduce map congestion.

In 2021, only the public schools had TRI facilities (*n* = 120) located within 1-mile of their buildings and a total of 100,517,930.40 pounds of regulated air, land, and water toxic releases. The public schools also had the most Brownfield/Superfund sites (*n* = 698) located within the buffer followed by private schools (*n* = 32) and then for-profit charters (*n* = 28). The not-for-profit charters had none of these sites within a 1-mile distance from them.

### Blood Lead Levels (BLLs)

**Fig. 2:**
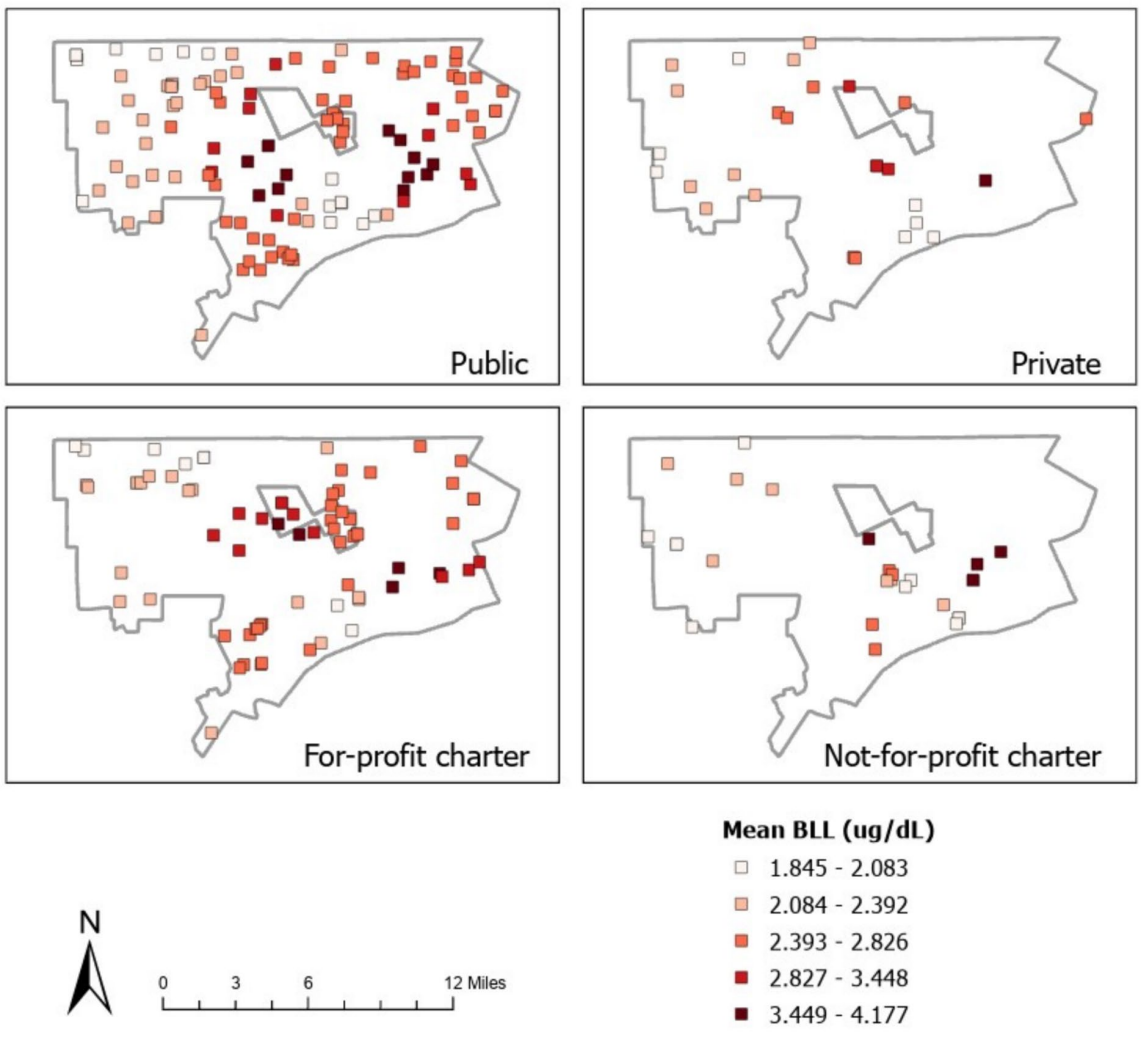
Mean BLL concentrations shown for each school of choice type as averaged within the 1-mile buffer

The Detroit Metropolitan Area (DMA) BLL screening rate (proportion of children BLL tested divided by the census 2006–2010 5-year estimate of the childhood population as a whole under 5 to 14 years) was 0.53. The one-way ANCOVA met normality check assumptions. There was a significant difference in mean BLL between school of choice type [*F*(225, 13) = 8.292, *p* ≤ 0.001] while controlling for enrollment. Post hoc tests showed that there was not a significantly different mean BLL within the 1-mile buffer across school of choice type controlling for enrollment. From the adjusted means, it is clear that the public schools most related to higher mean BLLs within the buffer (*M* = 2.622) followed by for-profit charters (*M* = 2.614), then not-for-profit charter (*M* = 2.508), and lastly private schools (*M* = 2.323). The partial Eta squared of 99.3% indicates the variance in school of choice type is explained by the mean BLLs within the buffer controlling for enrollment. For-profit charter schools followed closely by the public schools had significantly higher mean BLLs within the buffer than the not-for-profit charter and private schools. Figure 2 maps the mean BLL concentrations associated with the school of choice type.

Public schools had a range of 0–106 µg/dL BLL with a mean of 2.233 (*n* = 147,763), private schools 0–61 µg/dL with a mean of 1.954 (*n* = 3161), for-profit charters 0–63 µg/dL with a mean of 2.310 (*n* = 2867), and not-for-profit charters 1–11 µg/dL with a mean of 2.260 (*n* = 120).

### Neighborhood Segregation Indices

**Fig. 3:**
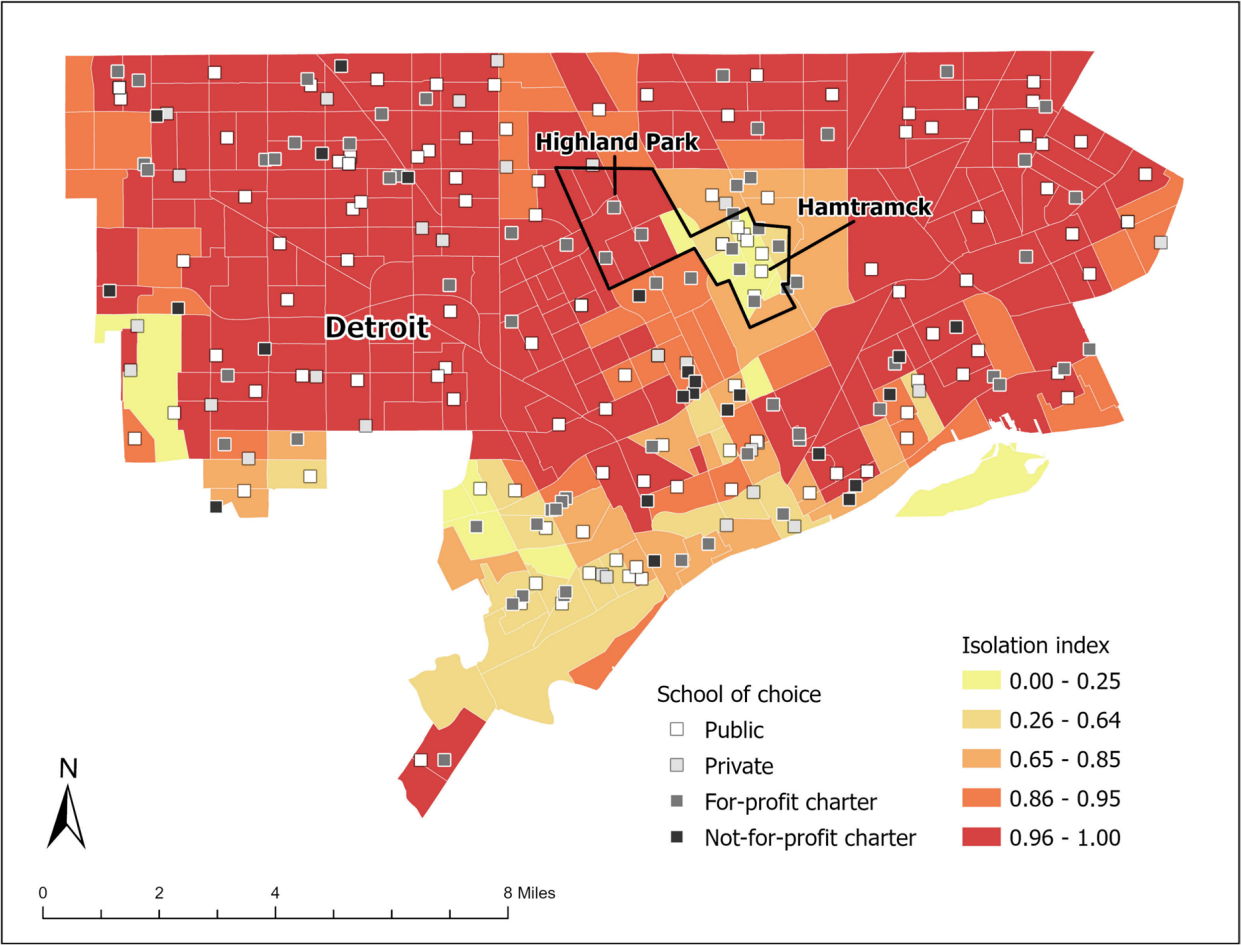
Segregation isolation indices for the cities of Detroit, Hamtramck, and Highland Park

**Fig. 4:**
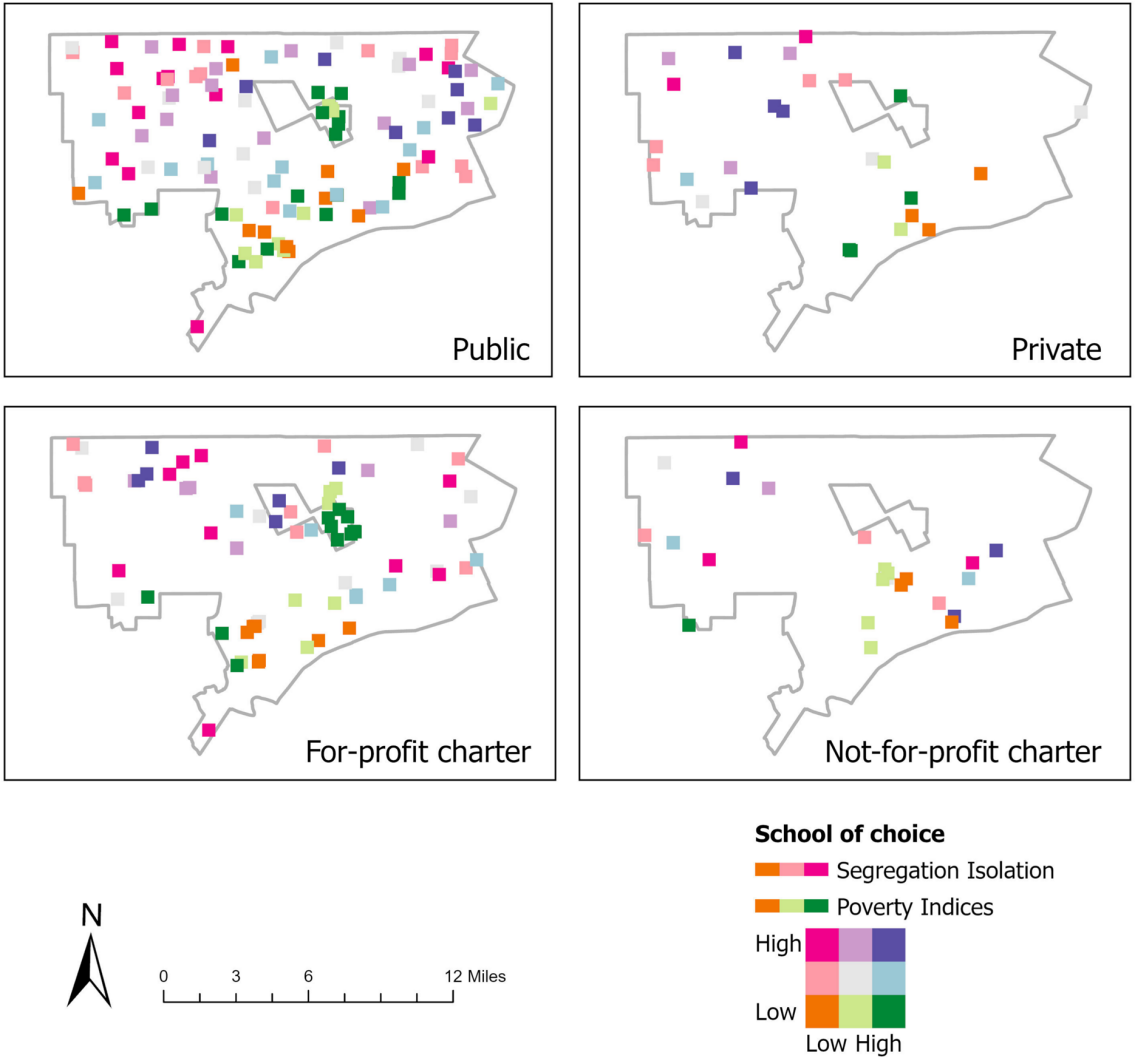
Levels of segregation using the isolation index and poverty indices for the cities of Detroit, Hamtramck, and Highland Park by each school of choice type

**Fig. 5:**
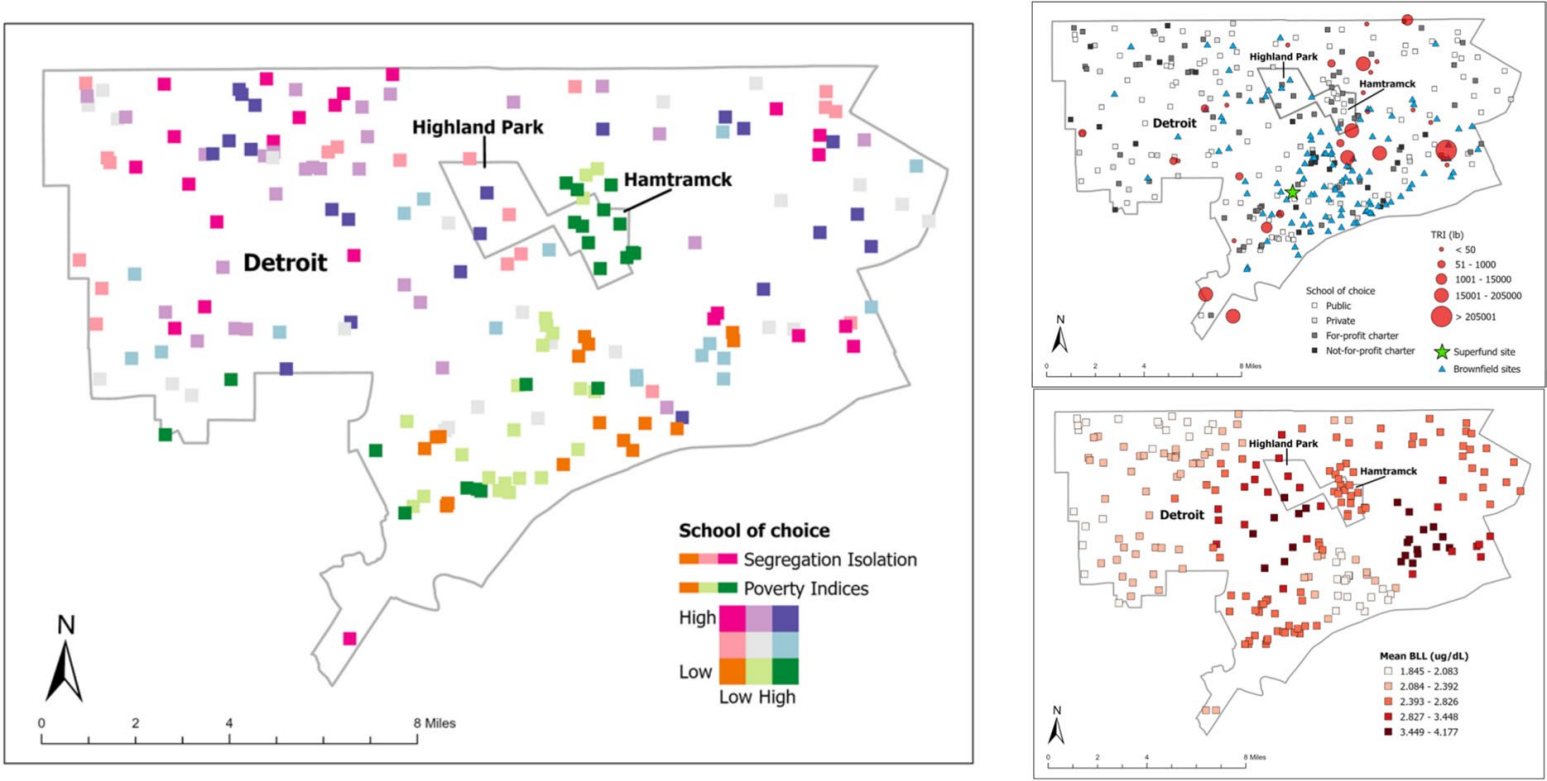
Side-by-side comparison of levels of segregation and poverty and locations of TRI facilities, Brownfield/Superfund sites and mean childhood BLLs for the cities of Detroit, Hamtramck, and Highland Park for all schools of choice types

Figure 3 displays the levels of segregation using the isolation index per census tract along with the school of choice type. Figure 4 shows levels of segregation and poverty indices by each school of choice type. Figure 5 combines all types of schools. The mean level of segregation for public schools was 0.87 (range, 0.15–0.92), for private schools, 0.86 (range, 0.48–0.93), for for-profit charter schools, 0.84 (range, 0.17–0.90) and for not-for-profit charter schools, 0.92 (range, 0.75–0.96). The mean level of poverty for public schools was 34.2% (range, 4.3–71.0%), for private schools, 26.7% (range, 4.3–45.9%), for for-profit charter schools, 37.3% (range, 11.0–65.7%) and for not-for-profit charter schools, 34.0% (range, 10.7–60.6%). Thus, all schools in Detroit, Hamtramck, and Highland Park were located in racially segregated and relatively high-poverty neighborhoods. Public schools and for-profit charter schools were located in a wide range of segregation and poverty levels—i.e., racially mixed to highly segregated neighborhoods with over a third of these neighborhoods being of high poverty. In contrast, private schools were generally located in highly segregated but less poor neighborhoods, and not-for-profit charter schools were located in highly segregated areas with a range of poverty levels.

Notable are the high poverty concentrations of Hamtramck public and for-profit charter schools and the high poverty and segregation of the for-profit charter schools located in Highland Park. Public and for-profit schools located in high concentrations of segregation, poverty and especially both, appear to align with areas dominated by TRI facilities, Superfund/Brownfield sites, and higher childhood BLLs. See Fig. 5 for a side-by-side comparison map.

## Discussion

This study found that for-profit charters, followed by public schools, provided the oldest buildings with the greatest potential exposure to a myriad of indoor toxicants. Public schools were located most closely to TRI and Superfund/Brownfield sites, potentially exposing their children to another set of pollutants. They were also located in the most blood-poisoned areas of the cities followed by for-profit schools. As introduced, this may be because public school areas of the cities have experienced disinvestment and their older neighborhood housing and location have been targets of Brownfield, TRI, siting, etc. For-profit charter schools might choose to locate schools on/in less expensive sites of abandoned industrial and commercial facilities.

Public schools are widely dispersed throughout the cities of Detroit and Hamtramck with the greatest range of segregation and poverty indices while for-profit charters are concentrated in the highest poverty neighborhoods. Grineski and Collins [11] found that public schools were located in the greatest Black-concentrated neighborhoods nationwide while this study found that not-for-profit charters and secondarily public schools were located in the most highly segregated neighborhoods. Like many studies [13, 14, 28], this study found that, primarily, for-profit charter schools are located in low-income areas. Mullen et al. [4], Gouge et al. [37], Grineski and Collins [11], and Kweon et al. [3] associated greater toxic school exposures with minority communities located in segregated Black communities.

Riel et al. [38] found that charter schools in general heighten school segregation and exacerbate racial and socioeconomic isolation compared to public schools. Farmer et al. [15] spatially associated Chicago’s new charter schools with Black neighborhoods and public schools of declining enrollment and closings instead of in more highly populated communities with overcrowded public schools, resulting in exacerbated financial stress on the school district. This may be the case with Highland Park’s all-for-profit charter schools replacing the public schools.

ArcGIS was used here as a powerful instrument to capture a variety of the spatial variables, join them, and then perform statistical analyses using those joined spatial relationships. A non-spatial analysis would not have captured all of the overlying predictors of exposure as they relate to pollution, segregation, poverty, and school of choice type.

Limitations of this investigation include a lack of data and facilities within the 1-mile buffer zone even though all pollutant facilities/indicators were encompassed. A model incorporating all school types as related to the Brownfield/Superfund and TRI sites with pounds of pollution was not possible given the low number/lack of facilities within the 1-mile buffer of the private, for-profit, and not-for-profit charters. As the buffer zone increased in radial distance, it did not improve the model. Increased buffer distances not only extended beyond the City of Detroit boundary but obscured individual potential exposure provided by each school as more overlap occurred and significance declined. Decreased buffer distances did not capture all of the variables. Another limitation is the assumption that the children attended the school closest to them. Given that Michigan is a school of choice state, students may have been driven to schools much outside their traditional school district. Another limitation is the use of building age as a proxy for exposure risk as opposed to obtaining actual inspection and renovation records that may have greatly reduced these risks to the children. Regarding childhood BLLs, public entities tend to analyze community-level exposures using means because they are more sensitive to skewed distributions typical of childhood BLLs. Knowing our population and that non-Hispanic Black, and Medicaid children have a higher percentage of BLLs than their counterparts [39] this study chose to conduct the analysis based on mean BLLs, especially given the large sample size. However, the median values may have been more sensitive to detecting outliers in this population.

This study hypothesized and showed that within the cities of Detroit, Hamtramck, and Highland Park, for-profit charter schools had the oldest school buildings and public and for-profit charter schools were located next to TRI industrial emissions, Brownfield/Superfund sites and fell within Black–White segregated impoverished neighborhoods associated with the highest BLL means, indicative of potential internal and external hazardous exposures. These indicators of toxic exposure have the potential to cause permanent long-term physiological and neurological damage such as cancer, asthma, headaches, dizziness, nausea, reduced IQ scores, slowed growth, hearing problems, brain damage, nervous system damage and behavior and attention problems needed to survive and be successful in school. This study shows that these outcomes would affect predominantly low-income/minority school-aged children. Public policy such as siting guidelines and school building maintenance should be used to decrease these potential exposure hazards and avoid worsening existing inequalities. The EPA provides voluntary guidance on school siting and roadway/pollutant exposure [40]. Rhode Island, California, Minnesota, Maryland, New Jersey, Washington, and New York require some type of environmental review before a school site is approved, such as requiring certain distances from toxic sites or meeting levels of remediation before building. Since 2004, seven Michigan bills have been introduced including prohibiting building new schools before an environmental assessment has been completed. All bills died in committee or failed to pass either the House or Senate. Introduction of these siting bills continues [41]. Research is growing in the direction that could inform policy, especially studies that explore environmental exposures and educational outcomes. These studies will enhance and address policies important to preventing further disadvantages to already vulnerable children.

## Data Availability

Data sets are available via request of the first author.

## Abbreviations

ANCOVA: Analysis of covariance
BLLs: Blood lead levels
CDC: Centers for disease control and prevention
EGLE: Environment, great lakes, and energy or Michigan’s environmental protection agency
EPA: Environmental protection agency
GIS: Geographic information systems
LEA: Local education agency
MDHHS: Michigan Department of Health and Human Services
PM: Particulate matter
TRI: Toxic release inventory
